# Should we recommend early overjet reduction to prevent dental trauma?

**DOI:** 10.1038/s41415-022-4916-0

**Published:** 2022-09-09

**Authors:** Martyn T. Cobourne, Andrew T. DiBiase, Jadbinder Seehra, Spyridon N. Papageorgiou

**Affiliations:** 4141510401001 Department of Orthodontics, Centre for Craniofacial and Regenerative Biology, Faculty of Dental, Oral and Craniofacial Sciences, King´s College London, London, UK; 4141510401002 Department of Orthodontics, East Kent Hospitals University NHS Foundation Trust, William Harvey Hospital, Ashford, UK; 4141510401003 Clinic of Orthodontics and Paediatric Dentistry, Centre of Dental Medicine, University of Zurich, Zurich, Switzerland

## Abstract

There is an association between increased overjet and risk of trauma to the maxillary incisor teeth in children and adolescents. It would therefore seem sensible to recommend overjet reduction as early as possible to help reduce this risk. However, orthodontic outcomes are essentially the same whether you start treatment in the early or late mixed dentition, while early treatment carries a heavier burden on compliance - taking longer and involving more appointments. This article explores the complex association between early overjet reduction and dental trauma in the context of current best evidence. Careful case selection is advised when justifying early intervention for increased overjet based on reducing trauma risk.

## Key points

- An increased overjet is significantly associated with higher odds of experiencing incisor trauma at all ages and stages of dental development.
- Early overjet correction can reduce the incidence of incisor trauma in children but there is wide variation in effect and the current evidence base is heterogeneous and at high risk of bias.
- Careful case selection for overjet reduction in the early mixed dentition is advised, focusing on those children with a significant increase in the overjet (>10 mm), excess maxillary incisor tooth show (short upper lip length, gummy smile, significant proclination) and those being teased or bullied at school because of their dental appearance.

## Introduction

Traumatic injuries to the dentition are a relatively common problem among children and young adults, with life-long consequences for affected individuals.^1^ The prevalence of dental trauma, which predominantly affects the maxillary incisor teeth, ranges from 10-12% at ages 15 and 12, respectively, in the UK.^2^ Global prevalence has been reported at just over 15% in the permanent dentition, with up to 18% of 12-year-olds affected.^3^ A wide range of risk factors are associated with dental trauma, including: patient sex; increased overjet (particularly with dental protrusion and inadequate lip coverage); anterior open bite; risk-taking children; certain medical disorders, such as epilepsy, cerebral palsy or learning difficulties; social deprivation; obesity; inappropriate use of the teeth; previous dental injury and oral piercings (Table 1).^4^^,^^5^^,^^6^^,^^7^^,^^8^

**Table 1:** Summary of selected factors associated with traumatic dental injuries, re-analysed with meta-analysis of odds ratios

Factor	Control group	Experimental group	Dentition	Studies	Odds ratio	95% confidence interval	P
Sex^*^	Female	Male	Primary	13	1.25	1.09-1.43	0.001
Lip coverage^**^	Adequate lip coverage	Inadequate lip coverage	Primary dentition (0-6 years)	10	1.88	1.36-2.60	<0.001
			Mixed/permanent dentitions (7-14 years)	20	2.37	1.66-3.39	<0.001
			Permanent dentition (12-19 years)	16	2.09	1.41-3.09	<0.001
Overjet^**^	Overjet ≤3 mm	Overjet >3 mm	Primary dentition (0-6 years)	4	3.08	2.41-3.94	<0.001
			Mixed/permanent dentitions (7-14 years)	11	1.94	1.44-2.61	<0.001
			Permanent dentition (12-19 years)	7	2.15	1.32-3.50	0.002
	Overjet ≤5 mm	Overjet >5 mm	Mixed/permanent dentitions (7-14 years)	12	2.02	1.50-2.72	<0.001
			Permanent dentition (12-19 years)	12	2.03	1.44-2.87	<0.001
Anterior open bite^**^	No anterior open bite	Anterior open bite	Primary dentition (0-6 years)	9	1.77	1.28-2.43	0.001
Previous trauma^†^	No previous trauma	Previous trauma	Pooled	4	2.79	1.80-4.33	<0.001
Early treatment (headgear or functional appliance)^‡^	Untreated (treated late)	Treated early	Pooled	4	0.48	0.28-0.81	0.006
Key: * = Data^5^ meta-analysed with REML random-effects meta-analysis ** = Data^8^meta-analysed with REML random-effects meta-analysis † = Data^7^ converted to odds ratios and meta-analysed with REML random-effects meta-analysis ‡ = Data^4^ supplemented by adding data^6^ and meta-analysed with REML random-effects meta-analysis							

**Fig. 1:**
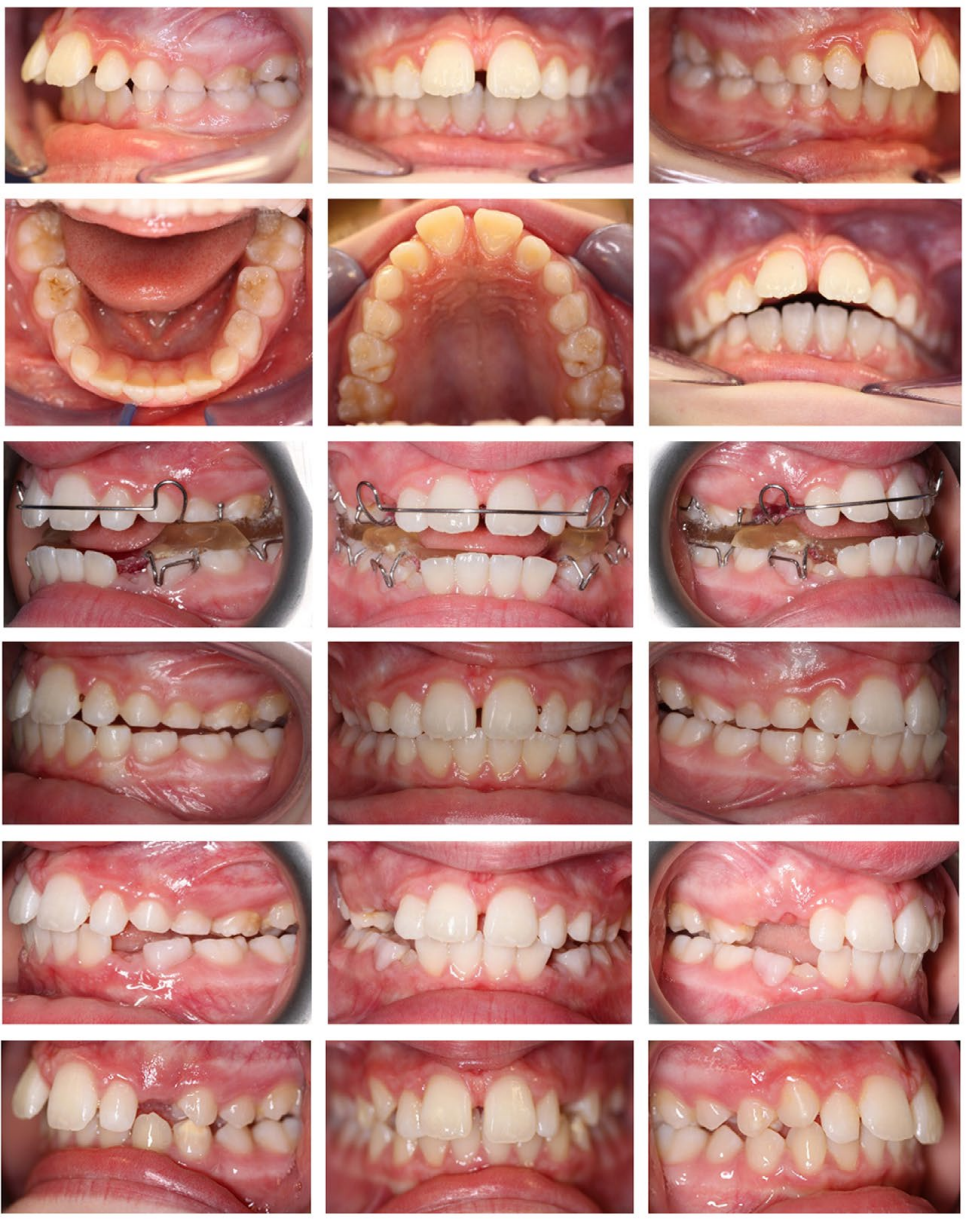
Treatment of a nine-year-old girl with a significant overjet in the mixed dentition with a twin-block functional appliance. Overjet correction took place very rapidly but a prolonged period of part-time appliance wear was required to maintain this while waiting for progression into the early permanent dentition and the placement of fixed appliances to detail the occlusion. Indeed, progressively reduced wear and ultimately, loss of the appliance, led to some relapse in the sagittal correction as she entered the permanent dentition

Among these risk factors, an increased overjet is significantly associated with higher odds of developing trauma at all ages and stages of dental development, with traumatic dental injuries attributable to a large overjet in 21% of cases globally.^9^ Children in the mixed or permanent dentitions (7-14 years) with an overjet >5 mm have 2 times the odds of experiencing a traumatic dental injury, while children in the permanent dentition (>12 years) with an overjet >5 mm have 2 times the odds compared to children with an overjet <5 mm.^8^ Given these data, it is important that preventative measures are considered at an early stage in children with an increased overjet to reduce the risk of dental trauma. These measures should include preventative advice and the use of mouth protection,^10^ particularly during contact sports, and ultimately overjet reduction with orthodontic treatment. An important question for the general dental practitioner (GDP) and orthodontist is whether to recommend early orthodontic treatment for overjet reduction specifically to reduce the likelihood of trauma. Although this would seem sensible, early treatment of Class II malocclusion is associated with some disadvantages; in particular, an increased overall treatment time, the need for prolonged retention of overjet reduction in the mixed dentition before a final phase of fixed appliance treatment once the child enters the permanent dentition and potential loss of compliance over the longer-term (Fig. 1). Moreover, the data relating to early overjet reduction and trauma prevention are complex and require careful scrutiny.

## Early correction of Class II malocclusion

It is inevitable that the practising GDP will see many children with an increased overjet during their working lifetime and if it is >6 mm, there is a defined treatment need in the UK.^11^ An increased overjet is often established well before the age of ten and it can be difficult to advise on the best time to intervene.^12^ The wider issues relating to early Class II correction have been debated among the orthodontic community for decades, with some of the first clinical studies demonstrating significant dental and skeletal changes in children with moderate to severe Class II discrepancies who undertook intensive treatment in the early mixed dentition.^13^ The advocates of early intervention claimed that starting at this time maximised success through enhanced skeletal effects, most notably using functional appliances and/or headgear. However, much of the data supporting these claims were retrospective, which invariably over-emphasised the positive effects of treatment.^14^ In addition, there had been more than a suspicion from some of these studies that the enhanced skeletal growth afforded by early treatment was often lost over the longer-term.^15^ Recognising this lack of high-quality evidence, three landmark randomised clinical trials (RCTs) were conducted over a decade in the early 2000s, two in the USA and one in the UK.^16^^,^^17^^,^^18^^,^^19^^,^^20^^,^^21^ These trials compared early mixed dentition treatment of Class II malocclusion with either a functional appliance (bionator or twin-block) and/or headgear, followed by any further treatment required in the permanent dentition, to a single course of comprehensive treatment carried out in early adolescence. More recently, another RCT based in Sweden has investigated the effects of early headgear-activator treatment in Class II children with excessive overjet.^6^ Collectively, the American and UK studies found that while early treatment is effective in reducing an increased overjet; at the end of the overall evaluation period, no clinically significant dental or skeletal differences are apparent between children treated early or late. These findings are consistent with the wider prospective literature on treatment of Class II malocclusion in children, suggesting few real advantages of early treatment.^4^

## Early treatment to prevent upper incisor trauma?

**Fig. 2:**
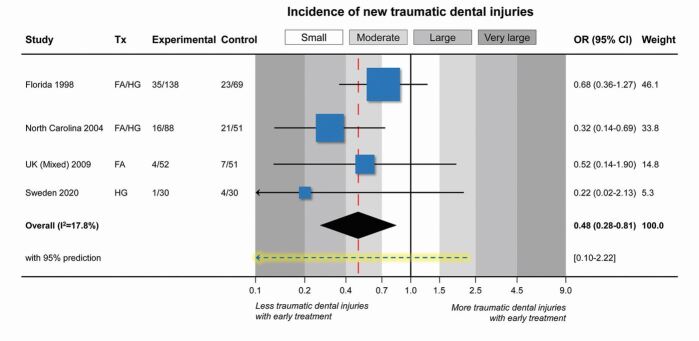
Forest plot depicting the pooled incidence of new incisor trauma from four RCTs investigating early versus late treatment for increased overjet (using a restricted maximum likelihood [REML] random-effects meta-analytical model). Data extracted^4^^,^^6^ and given as patients with trauma divided by overall patients in each group. Note the Sweden study has only reported data for the early phase of treatment. (FA = functional appliance; HG = headgear; OR = odds ratio; Tx = treatment)

Interestingly, these four trials have shown some association between early treatment and a reduction of new incisor trauma (Fig. 2). This is potentially important because it represents a good reason to consider undertaking overjet reduction earlier. In simple terms, the risk of incisor trauma was reduced by around a half (from 25.5% to 14.2%) in children having their overjet corrected early, but caution is advised when interpreting these results because there was wide variation in effect across trials (Fig. 2). The largest effect has been seen in the most recent Swedish trial; however, most of these 8-10 year old children had actually experienced their trauma before enrolment in the trial and therefore prevention through overjet reduction would have required starting treatment even earlier. In addition, it is unclear whether those experiencing trauma during the trial were new cases or repeat occurrences and this study is yet to report on final treatment outcomes (after the fixed appliance phase) for both randomised groups.^6^ It is also important to note that the Swedish study receives very little weight in the meta-analysis (5.3%) due to its small sample and low overall trauma incidence (8.3%, compared to 10.7% for the UK study;^17^ 26.6% for the North Carolina study;^22^ and 28.0% for the Florida study^23^) (Fig. 2). One possible reason for these variations is that none of these RCTs used trauma as their primary outcome (which would almost certainly require much larger sample sizes) and data collection relating to incisor trauma lacked specificity between trials. There were differences in how dental trauma was recorded and a lack of clinical detail in the classifications of trauma type and severity.^17^^,^^22^^,^^23^This might have influenced why the baseline trauma data seem to have changed with successive systematic review of this subject for three of the trials, despite the obvious binary nature of trauma incidence (it either happened or it did not).^4^^,^^24^ This has inevitably affected data analysis and interpretation and the current evidence should only be regarded as being of low to moderate quality.

## Treatment-timing decisions

So where does all this leave the GDP or orthodontist faced with a young child who has a large overjet? What does the best evidence tell us about the timing of treatment for this child and what advice should we be giving to patients and their parents? These studies do not say that early treatment should be carried out routinely in these children but they do demonstrate that there might be a potential difference in outcomes between those treated early or later. We should also consider some other factors. Apart from a higher risk of trauma, an increased overjet has been associated with a negative impact on oral health-related quality of life (OHRQL) and potentially makes a child more susceptible to victimisation and bullying,^25^^,^^26^ although early correction does not seem to influence OHRQL.^6^ Therefore, it would seem sensible to take a pragmatic approach and incorporate a key principle of evidence-based medicine - using your clinical judgement to do what is best for your patient within the context of the best available evidence. In some children, it would therefore seem prudent to consider early treatment, particularly if there is a perceived greater risk of dentoalveolar trauma or they are being teased because of very prominent teeth (Box 1). However, we need to be honest with our patients and not advocate early Class II treatment for all based upon the concept of achieving significantly enhanced alteration in facial growth or oral function, less need for adolescent premolar extractions, or indeed, a fundamentally better treatment outcome. It needs to be remembered that early treatment does place an increased burden on the patient, goes on for longer and involves more appointments with the orthodontist. All these factors need to be balanced and fundamental decisions about treatment-timing should be tailored to each individual patient. The evidence base on this subject is growing, but more work needs to be done.

Box 1Factors that might influence the decision to correct an increased overjet early

- Significantly increased overjet (>10 mm) or tooth show (short upper lip length, gummy smile, significant proclination)
- Patient is being bullied at school
- Female patient (entering the pubertal growth spurt earlier)

## Conclusions

This short review has highlighted the question of early orthodontic treatment and focused on the management of Class II discrepancies and risk of dental trauma. Although early treatment does not result in improved overall outcomes when compared to later treatment, some consideration should be given to starting early when it is thought that there is a real increased risk of dental trauma or a child is being teased because of their overjet.
